# The half-painted picture: Reviewing the mental health impacts of cancer screening

**DOI:** 10.1097/MD.0000000000030479

**Published:** 2022-09-23

**Authors:** Lauren P. Wadsworth, Inga Wessman, Andri Steinþór Björnsson, Gudbjorg Jonsdottir, Sigurður Yngvi Kristinsson

**Affiliations:** a University of Iceland, Reykjavik, Iceland; b University of Iowa Hospitals and Clinics, IA City, IA; c Landspiltai University Hospital, Reykjavík, Iceland.

**Keywords:** anxiety, cancer, cancer precursor, depression, mental health, oncology, PTSD, trauma

## Abstract

Cancer screening is recommended for select cancers worldwide. Cancer screening has become increasingly effective and accessible and often increases overall survival. However, the mental health effects of cancer screening, such as its impact on depression, anxiety, and post-traumatic stress disorder, are largely unknown. Conflicting available literature indicates the negative, neutral, and positive mental health effects of cancer screening across cancer types. There are a limited number of randomized controlled trials measuring the mental health effects of cancer screening. Overall, the more negative and life-threatening the screening results, the greater the mental health effects. Screening for cancer without a known precursor, for example, due to family history, can have positive impacts such as decreased worry and increased quality of life. However, receiving a cancer diagnosis often has negative mental effects that increase with the life-threatening potential of malignancy. In this study, we review the existing literature and provide recommendations for future research to determine if and when cancer screening is the best practice.

## 1. Introduction

Cancer screening has become an increasingly common practice because of the poor survival rates associated with many cancers. The mental health impact of cancer screening has been a growing conversation, as emerging studies have shown contradictory results regarding the mental impact of screening and have included negative, neutral, or positive impacts on mental health.^[1–7]^ Whether cancer screening is justifiable remains an empirical question. The clinical utility of large-scale screening procedures remains controversial.^[4,8–13]^ While extending survival rates is a clear argument for screening practices, there can be a number of harmful effects of cancer screening, including negative effects on patient mental health.

**Figure 1.**
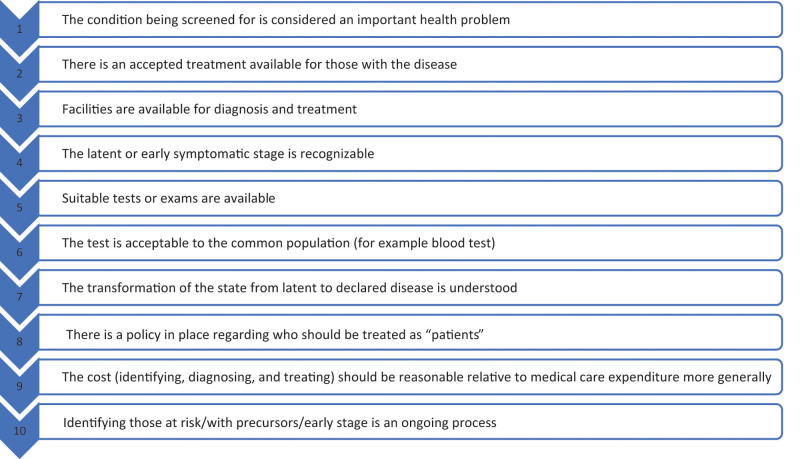
Wilson and Junger conditions for justifiable screening.

Wilson and Jungner^[14]^ proposed that screening programs are justifiable when ten conditions are met (Fig. 1). In their list, Wilson and Jungner do not mention an important aspect of screening: the lack of significant negative mental health impacts of undergoing screening and receiving a range of possible results (true positive, false positive, or negative result). Some guidelines mention that screening can have negative mental health impacts.^[15]^ but they do not specify when screening should or should not take place and how mental health symptoms arising from screening should be addressed.

It is vital to understand the full scope of positive and negative impacts of screening, including short- and long-term mental health effects. At present, our understanding of the mental health effects of screening is half painted. The available data are not comprehensive, as studies often lack rigorous methods or adequate sample sizes. Furthermore, available studies often use nonvalidated questionnaires and do not employ standardized measures of mental health symptoms. This review will explore the existing evidence of the effects of cancer screening on mental health across different screening protocols and malignancies, and will identify future research needs.

## 2. Evidence on the mental health effects of cancer screening

Despite advances in cancer screening, available data on the mental health effects of screening are limited. Each of these screening procedures likely has different effects on short- and long-term mental health, which must be understood as the ethics of cancer screening.

### 2.1. Mental health effects of screening in subjects without a known cancer precursor or risk factor

A number of studies have shown that cancer screening has little to no negative impact on mental health in the absence of a known precursor.^[1,16,17]^ A study investigating the impact of colonoscopies showed no impact on quality of life using the Short-Form Quality of Life Assessment (SF-36)^[18]^ in the short (2–3 hours) or moderate (1 month) term.^[16]^ The largest study (n = 21,944) on colorectal cancer screening found effects of receiving abnormal results on the Hospital Anxiety and Depression Scale (HADS)^[19]^ and Short-Form Health Related Quality of Life (HRQL) Form^[20]^ or by participating in screening in general.^[21]^ Those with healthy results showed improvements in all the measures.

Evidence suggests that screening can increase mental distress. A systematic review reported that cancer screening can cause low-to-moderate levels of distress.^[22]^ A systematic review of lung cancer screening found that the effects varied across studies, but in general, any negative effects of screening diminished or resolved at long-term follow up (≥6 months postscreening^[23]^).

Cancer screening can positively impact mental health. O’Neill et al^[24]^ (n = 102) found no evidence of short-term negative impacts of pancreatic screening on mental health, measured using 2 Likert-scale questions asking about worries about developing pancreatic or other cancers. At the year follow up, regardless of the screening results, participants reported significantly decreased worry about developing cancer. In a study (n = 231) investigating the effects of colonoscopies, 30% of participants reported a clinically significant improvement in the vitality and mental health domains of quality of life at 5 weeks, using the Short-Form Quality of Life Assessment (SF-36).^[17,18]^

Cancer screening may also promote healthy practices among the participants. In a study (n = 187) investigating mental health impacts (state-trait anxiety inventory (STAI), psychological consequences of screening [PCQ], and the HADS) of skin cancer screening (via skin examination), researchers found that screening had no negative impact on mental health 3 months after examination. Further, those who underwent more thorough skin examinations reported better skin cancer prevention methods postexamination and increased the likelihood of recommending exams to peers. Those with more thorough examinations also reported greater satisfaction with the exam.^[25]^

In summary, across different types of cancers, in the absence of a cancer precursor or risk, screening has a range of potential impacts (from neutral to positive to negative) on mental health. The positive mental health effects of screening are compelling, and alongside the potential for increasing positive patient behaviors, support screening in those without known precursors, given positive results on other endpoints, such as cancer survival. However, the findings should be considered alongside the methodological limitations. Frequently used mental health measures, such as the SF-36 and HADS, may be outdated, as they were created in the 1980s through the early 1990s and use colloquial phrases to assess mental health (“Have you been a happy person,” “I feel cheerful”) as opposed to symptoms correlated with the Diagnostic and Statistical Manual of Mental Health Disorders (DSM). Carefully designed studies with up-to-date measures are needed to fully understand the impact of cancer screening on mental health.

### 2.2. Mental health effects of screening in subjects with a known cancer precursor or risk factor

Evidence has shown that cancer screening can have a negative impact on mental health. One study (n = 2537) offered low-dose chest CT screening for participants at risk (current or former smokers considered at risk using a risk prediction tool) for developing lung cancer and showed that clinically significant anxiety, as measured using the State-Trait Anxiety Inventory (STAI), increases at 1 year follow up as a result of the screening. No differences in HRQL were found.^[26]^

High-risk individuals, such as those with a family history of pancreatic cancer, may benefit from cancer screening.^[27]^ One study (n = 198) investigated the effects of cancer screening in patients with a family history of pancreatic cancer or *BRCA2* mutation. Across 3 months, there was no effect of screening on general/global distress or risk perception, but those with a family history showed decreases in cancer-related worry.^[28]^

In a study exploring screening effects of a pancreatic cancer genetic counseling and screening intervention, 129 participants with a familial history of pancreatic cancer or BRCA2 mutation were assessed at baseline and included prescreening and a genetic counseling session, and 3- and 12-month follow-up postscreening.^[29]^ The results indicate that intrusive thoughts about cancer decreased significantly over time. Cancer-related worry decreased at 3 months and showed a small, significant increase at 12 months.^[29]^

Screening can cause mental distress, as screening for one’s family risk for ovarian cancer (n = 157 women) can cause elevated anxiety (STAI) and depressive symptoms (Center for Epidemiological Studies Depression Scale^[30]^), which increase along with the patient’s level of perceived risk.^[31]^ In a study comparing patients with a family history of pancreatic cancer (n = 378) to those without (n = 1528), those with a family history were more willing to engage in cancer screening than controls.^[32]^

Despite mixed findings, Cazacu et al^[27]^ concluded that the psychological benefits of cancer screening in high-risk individuals still outweigh the potential harm. Future studies should investigate the potential benefits and harm associated with decreased cancer worry and intrusive thoughts in at-risk individuals, as we do not know the effects of decreasing cancer worry. Decreased worry could potentially decrease motivation for health practices and attending screening appointments. Thus, the secondary effects of increased or decreased mental health symptoms resulting from screening should also be explored. Well-validated modern measures of anxiety and depressive symptoms should be employed in future studies.

### 2.3. Mental health effects of diagnostic risk factor

Some studies have suggested that screening positive for a cancer risk factor might not have a mental health impact. A recent cross-sectional study (n = 552) compared the severity of anxiety and depressive symptoms between individuals diagnosed with precursors to multiple myeloma (MM), monoclonal gammopathy of undetermined significance (MGUS) and smoldering multiple myeloma (SMM), individuals newly diagnosed with MM, and individuals in MM treatment. The study did not find a significant difference in anxiety symptoms between groups using the Generalized Anxiety Disorder 7-item scale (GAD-7). However, levels of depressive symptoms (Patient Health Questionnaire 9-item scale; PHQ-9) were higher for those in the MM treatment group compared to those newly diagnosed with MGUS. Patients with MGUS report higher physical HRQL (measured by the Short-Form General Health Survey 12 Item) than patients with newly diagnosed MM and treated MM.^[33]^ The results indicate that having MGUS versus MM has a similar impact on anxiety, but not depressive symptoms.

Being diagnosed with a precursor or genetic condition that increases the likelihood of cancer development has been associated with higher levels of anxiety,^[34-37]^ depression,^[38]^ and post-traumatic stress disorder (PTSD) symptoms,^[39-41]^ especially shortly after the results are obtained. A meta-analysis found that testing positive for BRCA1/2 is associated with a small increase in anxiety symptoms 1 month after being informed of the results. However, after 1 month, these symptoms subsided to baseline. Those who received results that they were not carriers had a decrease in anxiety symptoms up to a month after the results, which also returned to baseline by the 1-month follow up.^[37]^ Similarly, screening positive for a genetic mutation that causes hereditary nonpolyposis colorectal cancer (HNPCC) syndrome has been shown to cause a small short-term increase in anxiety symptoms that returned to baseline at the 1- and 6-month follow ups.^[34-36]^

Beran et al^[42]^ found that women with BRCA1/2 mutations (n = 117) reported significantly higher depressive symptoms (measured using the Center for Epidemiologic Studies Depression Scale) at 1 and 6 months after receiving the results. This pattern was maintained at the long-term follow up, 12 months after the results but was no longer significant. In a study investigating the impact of BRCA1 screening on those with a family history of BRCA1 (n = 287), there were no differences in depressive symptoms (HADS) 6-weeks after the results between those with and without BRCA1 compared to baseline.^[43]^ In this case, a positive BRCA1 result was not associated with an increase in depressive symptoms.

Screening results indicate that cancer precursors can also cause symptoms of post-traumatic stress disorder (PTSD). In a study by Hamann et al,^[6]^ carriers of BRCA1/2 mutations (n = 65) were more likely to experience symptoms of PTSD (measured via semistructured diagnostic interviews) resulting from the screening experience, compared to noncarriers, 3 to 6 months after being informed about BRCA1/2 status. This study was unique in that it measured psychological effects via semistructured interviews, which is more robust than most studies in this review. However, the sample size was small, only 12 of the participants had BRCA1/2, and only 3 of them developed PTSD symptoms after being informed of their status. Long-term follow-up data were unavailable. In a small qualitative study, patients diagnosed with MGUS (n = 14) reported poor psychological adjustment to their condition, particularly at the time of diagnosis. The participants also reported feelings of isolation, increased uncertainty, and limited psychosocial support.^[44]^

In summary, the mental health effects of receiving positive results of having a cancer precursor or risk factor seem more severe than those of less intensive screening. However, in general, negative effects seem most prominent in the days and weeks after the results and typically subside over time. Interventions such as informing patients of these potential short-term effects and short-term therapy to cope with the results should be investigated as ways to ameliorate these effects. Indeed, women who were screened for breast cancer and reported being satisfied with the social support of their providers throughout the screening process had lower levels of anxiety.^[45]^

### 2.4. Mental health effects of a false-positive cancer diagnosis

The effects of false-positive cancer diagnosis results are mixed. Some studies have shown no effect of false positives on mental health.^[46-48]^ In a large multicenter study (n = 2812) of patients with lung cancer, participants receiving false-positive results experienced no significant differences in anxiety or quality of life (STAI and HRQL) at 1 or 6 months posttest (most had their screening result at 1 month follow up, all had it by 6 months), compared to those with a negative result.^[49]^ A meta-analysis of false-positive mammography results found that females with false positives were not more likely to experience depressive symptoms than those with normal results.^[50]^

False-positive cancer screening results can increase symptoms of anxiety while patients are awaiting further examination. In a National Cancer Institute study (prostate, lung, colorectal, and ovarian; PLCO), a subset of participants (n = 432) was screened annually for prostate, lung, colorectal, and ovarian cancer. Participants who received abnormal screening results reported worsening HRQL in the short term but not in the intermediate term.^[51]^ In a sample of individuals screened for colorectal cancer (n = 301), those with abnormal results (n = 165) had an increase in anxiety (STAI) and doubtfulness about the decision to be screened, which decreased over time.^[52]^

Anxiety symptoms tended to decrease once false results were corrected. For example, in a breast cancer study (n = 285), a short-term increase in anxiety symptoms (HADS) was noted with false-positive screening results for breast cancer such that half (46%) of women experienced borderline to clinically significant anxiety symptoms while awaiting further examination, although the effect sizes were small. However, no difference was found at 3 and 12 months following the false-positive results compared with women with clear screening results.^[53]^ A number of studies on colorectal and ovarian cancer screening show that individuals reporting small but significantly higher symptoms of anxiety following a false-positive result subsided after being informed about the absence of cancer.^[7,54,55]^ In a lung cancer screening study (n = 400), a small increase was found in anxiety symptoms (STAI) from baseline measures after indeterminate and suspicious results. However, anxiety symptoms returned to baseline scores 6 months after screening for those with suspicious results and 12 months for those with indeterminate results.^[56]^

Receiving false-positive results can increase depressive symptoms. In a study investigating the effects of breast cancer screening, females (n = 128) with false-positive results reported higher levels of depressive symptoms (HADS) at the follow-up appointment than at baseline. These increased levels of depressive symptoms were maintained at the 3- and 6-month follow ups. Depressive symptoms were also higher in the false-positive group than in those with normal results at 6-month follow up, although the effect sizes were small.^[57]^ Women with false-positive screening results reported higher depressive symptom levels compared to women with normal screening results, initially after screening, a 1-week period between receiving the recall letter and attending the recall visit. However, false-positive females (n = 285) reported fewer depressive symptoms, based on the HADS, at 3 and 12 months following screening compared to females with normal screening results.^[53]^

Some evidence has been found regarding the long-term effects of false-positive results on mental health and behavior. A systematic review found that women who received false-positive mammography results were less likely to return for the next screening round. The authors concluded that the psychological impacts (worsened well-being, anxiety, and depressive symptoms) of receiving a false-positive result can last up to 3 years.^[58]^ Additional studies have replicated some of these results in women who received a false-positive result for ovarian cancer, finding that they showed increased cancer-related distress, which may reduce the likelihood of future screening.^[59]^ Similarly, a subset of the PLCO study (n = 432) found that participants who received abnormal results were less likely to adhere to procedures going forward than those who received normal results.^[51]^

In summary, in most cases, false-positive cancer diagnoses have short-term negative mental health effects. In addition to negative mental health impacts, receiving false-positive results followed by corrected results can affect patient behavior. These behavioral changes should be considered alongside the benefits of cancer screening and delivery of results. Finally, consistent with the previous sections, the measures used in the above studies were not the gold standard for measuring anxiety and depressive symptoms. Better measures in larger samples must be employed to fully understand the effects of false-positive results.

### 2.5. Mental health effects of receiving a cancer diagnosis

Cancer diagnosis is a well-established risk factor for poor mental health. A meta-analysis of 58 studies^[60]^ found that individuals with cancer reported more depressive symptoms than individuals in the general population, although the effect size was small. The prevalence of patients with a depressive disorder varies widely between individual studies (0–46%), which might be due to psychological health being differently impacted by the type of cancer.^[60–62]^ Indeed, patients with lung, hematological, and gynecological cancers report the highest depressive symptom levels, and patients with skin and prostate cancers report the lowest depressive symptom levels, perhaps reflecting varying prognoses and treatments.^[61]^

Cancer can cause PTSD symptoms because of its life-threatening nature.^[39–41]^ A meta-analysis of 25 studies revealed that 7% to 14% of cancer patients self-reported cancer-related PTSD symptoms. Furthermore, structured clinical interviews suggested that 13% of cancer patients had a lifetime prevalence of PTSD, with 6% currently having PTSD.^[63]^ Cancer stage had a significant effect on cancer-related PTSD rates. Rates were lower in patients with early stage (I–II) cancer to patients with advanced stage (III–IV) cancer, ranging from 4% to 12% and 11% to 31%, respectively.^[63]^ Those diagnosed at younger ages also had a greater risk of PTSD.

Cancer diagnoses can also have long-term effects. One study (n = 1154) showed that 6 months after diagnosis, 22% of cancer patients reported symptoms indicating clinical levels of anxiety and 21% 1 year after diagnosis, 13% had clinical levels of depression, and 9% had both clinically significant anxiety and depression at both time points.^[64]^

Patients with clinical levels of anxiety are lowest in prostate cancer (8%), skin cancer (12%), and gastrointestinal cancer (17%), and highest in gynecological cancer (28%), lung cancer (26%), and head and neck cancer (24%).^[61]^ One study (n = 5889) looking the 1 month prevalence of mental disorders in patients with cancer revealed that 32% had current psychological disorders.^[65]^ This study is 1 of the few to enlist diagnostic interviews and has a large sample size. Unfortunately, no baseline assessments were performed.

In a breast cancer study, almost half of the women (n = 303) met the criteria for a psychiatric disorder.^[66]^ The most prevalent disorder in both groups was mood disorder, followed by anxiety disorder. Symptoms of depression were significantly associated with attitudes towards helplessness, hopelessness, fatigue, and a history of depression. These patterns of depression and anxiety have been replicated across studies and tend to peak in the short term after diagnosis and decrease over time.^[65,67,68]^ In cases of higher and/or persistent depressive symptoms, women report a lower quality of life and worse recovery.^[69]^

The mental health impacts of cancer diagnosis can be more imminent and life threatening than the cancer itself, causing psychological distress that can lead to suicide.^[70–74]^ In a large population-based study (n = 4722,099), patients with cancer had a 20% increased risk of suicide compared with the general population. Patients with pancreatic, mesothelioma, esophageal, and lung cancers are at the highest risk.^[75]^ A cohort study (n = 6073,240) investigating immediate suicides after a cancer diagnosis found that those with a cancer diagnosis were at an increased risk of dying by suicide within the first week postdiagnosis (12.6%) or year (3.1%) compared to those without a cancer diagnosis.^[76]^ Another study (n = 24,489) found that suicide risk was the highest 2 months after multiple myeloma and acute myeloid leukemia diagnosis and was 3 times more frequent in male participants.^[77]^ These, and other findings in this section, call for increased psychological support for cancer patients, especially in the first 6 months after diagnosis. A meta-analysis on the impact of interventions on depression symptoms in cancer patients (n = 1362) found that targeted interventions can effectively decrease depression symptoms.^[29]^

In summary, the results of studies on the psychological impact of receiving a cancer diagnosis can increase symptoms of anxiety and depression and can cause PTSD. Cancer diagnosis can have long-standing effects, likely due to the life-threatening nature of some malignancies. However, withholding a diagnosis of cancer is not ethical. Instead, the differential impacts of screening on mental health should be considered relative to the severity of the cancer being screened, and the benefits of sharing results when false positives are possible must be weighed.

### 2.6. Summary of the existing literature

Taken together, literature on the mental health effects of cancer screening is limited (Table 1). Overall, the risk for increased mental health symptoms increases along with the risk and life-threatening nature of cancer. For example, screening individuals without any known precursors and with only a family history of cancer can have minimal impacts that resolve within months. Screening individuals with a known cancer precursor can increase anxiety, depression, worry, and intrusive thoughts; however, these effects usually return to baseline. Receiving a diagnosis of cancer can lead to significant increases in anxiety, depression, post-traumatic stress disorder, and suicide (Table 2). Interpreting these results alongside the risk of impairment or death due to cancer is a complex task and should include an interdisciplinary approach.

**Table 1:** Brief summary of mental health impacts of cancer screening.

Mental health impacts of screening procedures			
Screening procedure without a known precursor	Screening with a known cancer precursor or risk factor	Receiving a false-positive cancer result	Receiving a cancer diagnosis
• Minimal negative impacts that diminish within month	• Potential increase in anxiety and depression, which may return to baseline	• Short-term negative impacts (increased anxiety and depression) that are eliminated when results are corrected	• Negative mental health effects (increased anxiety, depression, PTSD symptoms)
• Can reduce worry and increase quality of life	• Could decrease worry and intrusive thoughts		• Increased risk of suicide

**Table 2:** Notable studies investigating the mental health impacts of various levels of cancer screening procedures.

Cancer type/test	Number of subjects or studies	Outcome measure	Key finding	Year, author, reference
Mental health effects of screening in subjects without a known cancer precursor or risk				
Colon	21,944	Hospital Anxiety and Depression Scale, Short-Form Health Related Quality of Life Form	No impacts on mental health by the screening procedure or receiving abnormal results	2016; Kirkøen et al^[21]^
Colon	231	Short-Form Quality of Life Assessment	30% of participants reported a clinically significant improvement of the vitality and mental health domains	2006; Taupin et al^[17]^
Breast, colorectal, prostate, lung, and cervical	13 observational trials; 9 RCTs	Most common measure was State-Trait Anxiety inventory	Mental health impacts were low, aside from in colorectal screening	2017; Chad-Friedman et al^[22]^
Mental health effects of screening in subjects without a known cancer precursor or risk				
Pancreatic	102	Cancer worry scale	People with a risk factor showed greater worry about developing cancer prior to screening. Screening resulted in no greater increase, and long-term positive benefits of mental health were evidenced.	2020; O’Neill et al^[24]^
Lung	2537	State-Trait Anxiety Inventory; Health Related Quality of Life	Those at risk had increased anxiety at 1 year follow-up. No differences on quality of life.	2019; Taghizadeh et al^[26]^
Pancreatic	129	Intrusive thoughts and worry about cancer	Intrusive thoughts about cancer decreased at 3 and 12 months post screening. Cancer worry decreased at 3 months and increased slightly at 12 months.	2012; Hart et al^[29]^
*Mental health effects of diagnosis of cancer precursor or risk factor*				
Multiple myeloma	552	Generalized Anxiety Disorder 7-item scale; Patient Health Questionnaire 9-item scale; Short-Form General Health Survey 12 Item	Patients in treatment for multiple myeloma reported higher depression and lower quality of life than those diagnosed with a precursor. No difference in anxiety levels between groups.	2019; Maatouk et al^[33]^
BRCA1/2 gene	65	semistructured diagnostic interview	BRCA1/2 carriers were more likely to report PTSD symptoms as a result of screening compared to noncarriers.	2005; Hamann et al^[6]^
BRCA1/2 gene	117	Center for Epidemiologic Studies Depression Scale	Females with the BRCA1/2 mutation reported significantly higher depression at 1 and 6 months after results. The pattern held at 12 months but was not significant.	2008; Beran et al^[38]^
Mental health effects of false-positive cancer diagnosis				
Lung	2812	State-Trait Anxiety Inventory; Health Related Quality of Life	Participants receiving false-positive results experienced no significant differences in anxiety or quality of life compared to those with a negative result (measured at 1 and 6 months)	2014; Gareen et al^[49]^
Prostate, lung, colorectal, and ovarian; PLCO	432	Health Related Quality of Life	Participants who received abnormal screening results reported worsening health related quality of life in the short term, but not intermediate term	2004; Taylor et al^[51]^
Colorectal cancer	301	State-Trait Anxiety Inventory	those with abnormal results (n = 165) had an increase in anxiety and doubtfulness about the decision to be screened, which decreased over time- especially once results were corrected	2014; Bobridge et al^[52]^
Breast	285	Hospital Anxiety and Depression Scale	A short-term increase in anxiety symptoms was associated with false-positive screening results for breast cancer, though effect sizes were small. No difference was found 3 and 12 months following the false-positive results compared to women with clear screening results	2001; Lampic et al^[53]^
Breast	128	Hospital Anxiety and Depression Scale	Those with false-positive results reported higher levels of depressive symptoms at the follow-up appointment, compared to baseline. These increased levels of depressive symptoms were maintained at 3- and 6-month follow-ups.	2012; Hafslund et al^[57]^
Mental health effects of a cancer diagnosis				
Meta-analysis including breast, lung, ovarian, pancreatic cancer and more	58 studies	Varied	Those with cancer slightly more likely to report depression than general population. Those with less life-threatening cancers reported less depression.	1997; van’t Spijker et al^[60]^
Meta-analysis	25 studies	Varied	PTSD symptoms more common in those with cancer diagnosis. PTSD symptoms were higher in those with more advanced stage cancers.	2015; Abbey et al^[63]^
Any type	1154	Hospital Anxiety and Depression Scale	People with cancer report higher levels of clinically significant anxiety and depression 6 and 12 months after diagnosis, with anxiety decreasing between timepoints and depression staying consistent.	2013; Boyes et al^[64]^
Any type	5889	Standardized computer-assisted Composite International Diagnostic Interview for mental disorders adapted for cancer patients	32% of people with a cancer diagnosis had a current mental disorder.	2014; Mehnert et al^[65]^
Any type	4,722,099	Mortality/Suicide Risk	Patients with cancer are at 20% increased risk of suicide compared to general population.	2012; Henson et al^[75]^
Any type	6073,240	Immediate suicide following cancer diagnosis	Those with a cancer diagnosis were at increased risk of dying by suicide within the first week postdiagnosis (12.6%) or year (3.1%) compared to those without a cancer diagnosis	2012; Fang et al^[76]^

### 2.7. The needed next phase: randomized controlled trials

To fully understand the mental health effects of cancer screening, future studies must adopt a strategic and thorough approach. Studies should emphasize comprehensive psychological assessments using well-validated assessments at baseline, short-term, and longitudinal follow-up, comparing groups with and without screening, and within the screened group, with negative, positive, and false-positive results. Further, researchers should ensure that when negative psychological effects are present, the effect sizes are large enough to warrant information on best practices. Most of the available literature has only been cross-sectional, focused on short-term mental health effects, and lacks a longitudinal follow-up.

There is a dearth of randomized controlled trials (RTCs) that specifically seek to answer the following question: what types of cancer screening are ethical? RCTs are needed to assess the effects of differential screening outcomes. Measures of mental health can easily be integrated into existing RCTs and studies investigating cancer progression. The field would benefit the most if RCTs employed common, validated measures of mental health and well-being. Indeed, a recent systematic review of the impact of cancer screening on mental health found that screening-associated distress was only detected in studies that used validated measures and was not detected using nonvalidated measures.^[22]^ Most studies only measured short-term distress (2 weeks before screening-1 month postscreening), underscoring the need for longitudinal studies. A more comprehensive assessment of mental health would provide a broader understanding of the role of cancer screening on functioning. Furthermore, differences across demographic features (age, race, ethnicity, gender, etc) must be explored. Integrating brief, easy-to-administer assessments into RCTs is a minimal investment for an exponential benefit. Having a thorough understanding of the mental health effects of cancer screening alongside physical and longevity gains will help us understand ethical screening types.

## 3. Conclusion

In summary, there is evidence of both the negative and positive effects of cancer screening on mental health. The need for reliable cancer screening procedures to trigger early intervention is critical not only to add years to life, but also to life to years. However, the speed of cancer screening research has been fueled by the urgency to save lives. Despite the importance of improving survival rates, methodically rigorous studies are required to determine their beneficial and harmful effects. Benefits such as prolonged survival and positive mental health impacts need to outweigh the potential harm and health costs.^[3,78,79]^ In-depth evidence on the mental health effects of cancer screening can help advocate for interventions, as research suggests that social and healthcare professional support can reduce anxiety levels.^[44,45]^ Specifically, carefully designed RCTs are needed to fully evaluate when screening is justifiable to complete a half-painted picture. RCTs can be strengthened by integrating validated mental health measures at baseline and longitudinally across groups. Integrating measures of mental health into RCTs will provide a deeper understanding of the likely intricate and changing effects of cancer screening on mental health over time.

## Author contributions

Lauren: conception, primary writer

Inga: conception, secondary writer

Andri: conception, editing, review, writing contributions

Sigurdur: conception, editing, review, writing contributions

Gudbjorg: editing, review, writing contributions
